# Endocrinological aspects of pituitary adenoma surgery in Europe

**DOI:** 10.1038/s41598-022-10300-1

**Published:** 2022-04-20

**Authors:** David Netuka, André Grotenhuis, Nicolas Foroglou, Francesco Zenga, Sebastien Froehlich, Florian Ringel, Nicolas Sampron, Nick Thomas, Martin Komarc, Mikuláš Kosák, Martin Májovský

**Affiliations:** 1 Department of Neurosurgery and Neurooncology, First Faculty of Medicine, Charles University and Military University Hospital, U Vojenske Nemocnice 1200, 169 02 Prague 6, Czech Republic; 2 Department of Neurosurgery, Radboud University Medical Centre Nijmegen, Nijmegen, The Netherlands; 3 1St Department of Neurosurgery, Aristotle University of Thessaloniki, Thessaloniki, Greece; 4 Department of Neuroscience “Rita Levi Montalcini”, Neurosurgery Unit, University of Turin, Turin, Italy; 5 Department of Neurosurgery, Lariboisière University Hospital, Paris, France; 6 Department of Neurosurgery, Johannes Gutenberg-Universität Mainz, Mainz, Germany; 7 Neurosurgery Department, University Hospital Donostia, San Sebastian, Spain; 8 Department of Neurosurgery, Kings College, London, UK; 9 Institute of Biophysics and Informatics, First Faculty of Medicine, Charles University, Prague, Czech Republic; 10 Department of Methodology, Faculty of Physical Education and Sport, Charles University, Prague, Czech Republic; 11 Department of Internal Medicine, First Faculty of Medicine, Charles University and Military University Hospital, Prague, Czech Republic

**Keywords:** Pituitary diseases, Diseases of the nervous system

## Abstract

Hormone-secreting adenomas are treated in many neurosurgical centers within Europe. The goal of the survey is to understand variance in practice management of pituitary tumors amongst neurosurgical centers. A list of departments performing pituitary surgery was created. The survey consisted of 58 questions. This study focuses on neurosurgical care of hormone-secreting adenomas. For analysis, the departments were divided into four subgroups: academic/non-academic, high-volume/low-volume, “mainly endoscopic/mainly microscopic practice” and geographical regions. Data from 254 departments from 34 countries were obtained. Most centers surgically treat 1–5 hormone-secreting adenomas per year. In prolactinomas this is the case in 194 centers, (76.4%), in GH-secreting adenomas: 133 centers, (52.4%), ACTH-secreting adenomas: 172 centers, (69.8%). Surgery as a primary treatment of prolactinomas is considered in 64 centers (25.2%). In 47 centers (18.8%), GH-secreting microadenomas are often treated pharmacologically first. Debulking surgery for an invasive GH-secreting adenoma in which hormonal remission is not a realistic goal of the surgery and the patient has no visual deficit surgery is always or mostly indicated in 156 centers (62.9%). Routine postoperative hydrocortisone replacement therapy is administered in 147 centers (58.6%). Our survey shows that in most centers, few hormone-secreting adenomas are treated per year. In about 25% of the centers, prolactinoma surgery may be regarded as first-line treatment; in about 20% of the centers, medical treatment is the first-line treatment for GH-secreting adenomas. Pretreatment for ACTH-secreting adenomas is routinely used in 21% of centers. This survey may serve as plea for neurosurgical care centralization of hormone-secreting adenomas.

## Introduction

Pituitary adenoma surgery is a field in which neurosurgery and endocrinology closely cooperate in patients’ management. Neurosurgeons are often involved in perioperative patient care, including patient counseling, blood sampling and hormone replacement (e.g., corticosteroid replacement). Basic knowledge of pituitary endocrinology is therefore inevitable for neurosurgeons.

We lack European data from neurosurgical centers, which treat hormone-secreting pituitary adenomas and perform perioperative endocrinological care. This study aims to shed light on different endocrinological aspects of pituitary adenoma surgery. We created an international investigational team of neurosurgeons and conducted an online survey among European departments.

## Material and methods

All 39 European member societies of EANS were approached. Each society was asked to distribute an online survey using the SurveyMonkey platform to their neurosurgery departments. Our goal was to cover a large number of neurosurgical departments in Europe that perform pituitary adenoma surgery. We created a list of qualified departments based on cooperation with EANS, national neurosurgical societies and personal communication with local neurosurgeons. Each member of the study group approached their national peers with a request to participate in the survey. Direct mailing to the Department of Neurosurgery chairpersons in Europe was conducted to achieve the highest possible coverage. Finally, each member of the study group covered his country. DN and MM were responsible for countries not covered by the remaining members of the study group. The goal was to enroll pituitary centers of excellence and all departments performing pituitary adenoma surgery.

The request was to complete the survey (https://www.surveymonkey.com/r/eu-pass) by the respective departments' chairpersons or pass it to a neurosurgeon in charge of the pituitary program. The target was one completed survey per department.

The survey consisted of 58 questions, which could be divided into three sections:

A. Demographics (countries, caseload per center, surgeons involved in pituitary surgeries, residents engaged in pituitary surgeries, pituitary boards)
B. Treatment of non-functioning adenomas.
C. Treatment of hormone-secreting adenomas (prolactinomas, growth hormone (GH)-secreting adenomas, adrenocorticotropic hormone (ACTH)-secreting adenomas)
D. Techniques (navigation, intraoperative imaging, technique of the surgery, postoperative MRIs, follow-up)

All methods were carried out in accordance with relevant guidelines and regulations.

This paper examines questions in section C, i.e. endocrinological aspects of pituitary surgery and perioperative patient care. The study period started on 1 April 2019 and closed on 30 June 2019. The remaining sections were covered in previous papers^1,2^. All questions used in survey are presented as supplementary data.

The minimum time to complete the survey was 12 min (min) (mean time 18 min). All received surveys were complete. Data from 34 European countries were obtained. Altogether, 254 European Departments of Neurosurgery completed the survey. Most responses came from Germany (60), Italy (28), France (22), the UK (16), the Czech Republic (13) and Spain (10).

A univariate analysis was planned based on the following variables:

- Academic/non-academic centers (based on participant answers)
- High-volume/low-volume centers. We defined a high-volume center as a center where > 30 surgeries for pituitary adenomas are performed per year^3^.
- Technique applied in the center: “mainly endoscopic” (> 90% of surgeries were performed endoscopically), “mainly microscopic” (> 90% microscopically and > 90% microscopically endoscope-assisted) and “mixed practice”.
- Regions: according to United Nations Statistics Division^4^.

### Statistics

Basic descriptive statistics were performed that included counts and percentages for each survey question. Differences in responses to survey questions based on selected grouping variables (academic center, surgical technique, case volume per year and region) were examined using Pearson’s chi-square test (Fisher’s exact test was applied as appropriate) with adjusted residuals. The level of statistical significance was set at α = 0.05. The descriptive statistical analysis (frequencies and percentages) and data processing were done with SPSS *statistical software (SPSS version 25*·0, SPSS Inc., Chicago, IL, USA) and Microsoft Excel.

## Results

### Prolactinomas

**Figure 1:**
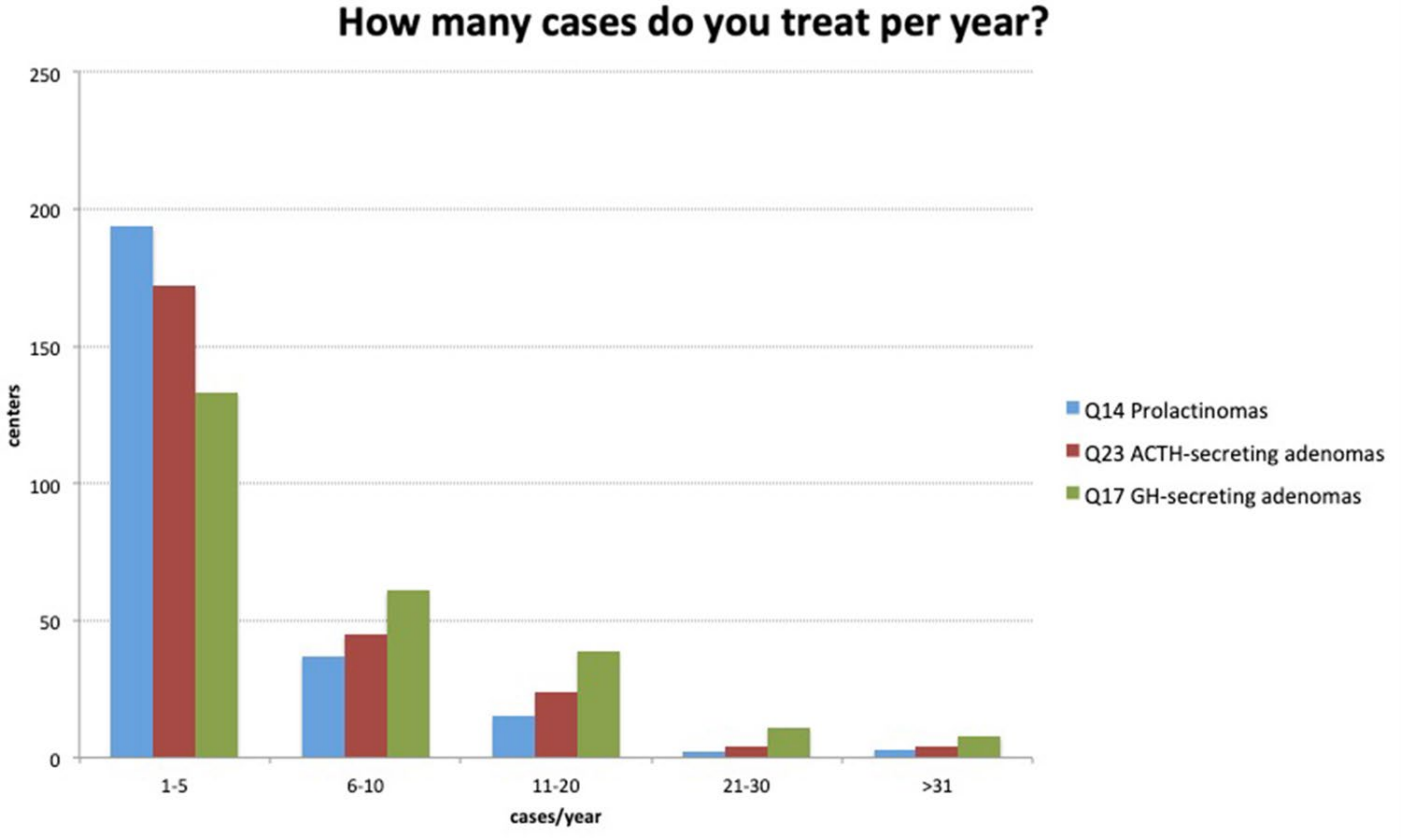
Hormone-secreting adenomas treated per year.

Most centers surgically treat 1–5 prolactinomas per year (194 centers, 76.4%) and only 7.9% of the centers treat > 11 prolactinomas per year. These data are summarized in Fig. 1.

We then asked about the time between consultation and surgery in patients with prolactinoma on medication, sudden apoplexy, severe headaches and moderate visual field deficit. Surgery as a primary treatment modality for prolactinomas is considered in 64 centers (i.e. 25.2%). In patient with apoplexy, surgery is performed on the same day in 55 centers (21.7%), within 48 h in 128 centers (50.4%) and within 7 days in 27 centers (10.6%). In 40 centers (15.7%), a wait-and-watch policy is first applied; next, medical therapy is initiated; and finally, a decision is taken.

### GH-secreting adenomas

Again, most centers treat 1–5 GH secreting adenomas (133 centers, 52.4%). Some 61 centers (24%) treat 6–10 GH-secreting adenomas and 19 centers (7.4%) treated > 20 adenomas per year (Fig. 1). In 47 centers (18.8%), GH-secreting microadenomas are often treated pharmacologically first (in ≥ 70% cases). Surgery is indicated as second-line treatment in 135 centers (53.3%). In GH-secreting macroadenomas, primary medical treatment is frequently performed in 49 centers (20%) and seldom in 144 centers (58.5%). Neurosurgeons were asked which medication was used for initial treatment—summarized in Table 1.

**Table 1:** Medication used for GH-secreting adenomas initial treatment (according to neurosurgeons).

Choices	Responses (%)
Dopamine agonists	26.3%
First-generation somatostatin analogs (lanreotide and octreotide, etc.)	55.4%
Second-generation somatostatin analogs (pasireotide, etc.)	21.5%
Growth hormone receptor antagonist (pegvisomant)	17.1%
N/A	30.3%

In 170 centers (67.5%), the whole surgical team and patient are informed beforehand whether a hormonal remission was a realistic surgical goal. On the other hand, debulking surgery for an invasive adenoma in which hormonal remission is not a realistic goal of the surgery and the patient has no visual deficit surgery is always indicated or mostly indicated in 156 centers (62.9%) and rarely or never in 39 centers (15.7%).

### ACTH-secreting adenomas

As in acromegaly, most centers treat 1–5 Cushing’s disease patients (172 centers, 69.8%). Some 45 centers (18%) treat 6–10 ACTH secreting adenomas and 8 centers (3.2%) treat > 20 adenomas (Fig. 1). In 53 centers (21.6%), pretreatment by ketoconazole or metyrapone is often used (in ≥ 70% of cases). However, in 136 centers (55.5%), this strategy is rarely or never used (in ≤ 20% of cases). Table 2 summarizes the rationale for pretreatment.

**Table 2:** The rationale for ACTH-secreting adenomas pretreatment (according to neurosurgeons).

Reason for pretreatment	Responses
Safer anesthesia	24.5%
Better postoperative healing	13.7%
Lower risks of postoperative medical complications	32.9%
To overcome the waiting period for surgery	27.7%
N/A	41.4%

Dynamic MRI is routinely used (in ≥ 70% of cases) in the diagnostic algorithm for Cushing’s disease in 121 centers (49.1%) and rarely or never used (in ≤ 0% of cases) in 85 centers (34.5%). 7 Tesla MRI is never used in a diagnostic algorithm in 234 centers (94.3%). Only in two centers (0.8%) is 7 Tesla MRI a routine procedure. Catheterization and petrosal sinus blood sampling is routinely performed in 17 centers (6.9%) and rarely or never in 192 centers (78.1%). If postoperative cortisol levels are inappropriately high, early reoperation in Cushing`s disease is considered in 138 centers (55.6%).

Other hormone-secreting adenomas were not included into our survey.

### Postoperative hydrocortisone substitution

Routine postoperative hydrocortisone replacement therapy is administered in 147 centers (58.6%). In addition, hydrocortisone is initiated in selected cases based on postoperative cortisol levels and primarily by the decision of a neurosurgeon in 49 centers (19.5%). In 55 centers (21.9%), the selective hydrocortisone substitution is managed by the endocrinologist.

### Academic versus non-academic centers

Some 88.2% of the non-academic and 74.5% of the academic centers treat 1–5 prolactinomas (*p* = 0.05). Patients with prolactinoma on medication with sudden apoplexy presented with severe headaches and moderate visual field deficit are more often treated within 48 h in academic centers (54.0% versus 37.7%, *p* = 0.05). More academic centers treat 6–10 GH-secreting adenomas per year than non-academic centers (27.5% versus 11.5%, *p* = 0.01). Altogether, 59% of the academic centers and 38.9% of the non-academic centers use the first-generation somatostatin analogs (lanreotide, octreotide) to treat GH-secreting adenomas (*p* = 0.01). Debulking surgery for an invasive GH adenoma in which hormonal remission is not a realistic goal of the surgery and the patient has no visual deficit is always performed in 27.0% of the academic centers and in 7.7% of the non-academic centers (*p* = 0.01). We asked what the goal of pharmacological pretreatment in Cushing's disease is. Some 31% of the academic centers and 13% of the non-academic centers report that pretreatment is used to overcome waiting for surgery (*p* = 0.01).

### Mainly endoscopy/mainly microscopy

Medical pretreatment of Cushing's disease is indicated to reduce the risk of postoperative complications in 39.4% of the endoscopic centers and 22.2% of the microscopic centers (*p* = 0.05). No other significant differences were observed.

### High- versus low-volume centers

**Figure 2:**
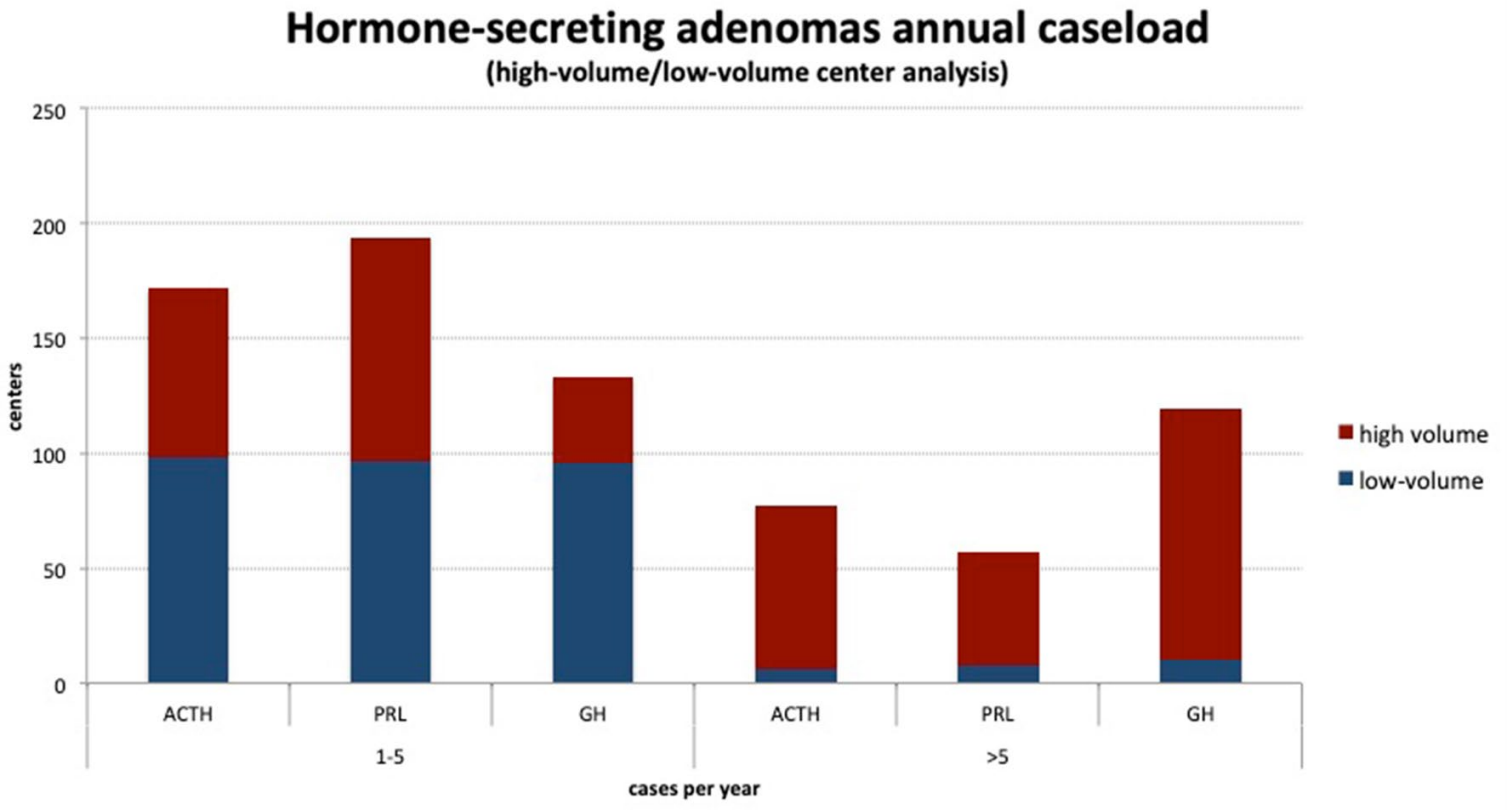
Hormone-secreting adenomas treated in high- and low-volume centers.

Expectedly, hormone-secreting adenomas are more often treated in high-volume centers (significant at 0.001 in all cases, Fig. 2).

In 76.7% of the high-volume and 61.9% of the low-volume centers, medical pretreatment of GH-secreting microadenomas is rarely or never done (0–30% of the cases) (*p* = 0.05). If medical treatment in GH-secreting adenomas is provided, first-generation somatostatin analogs are used in 60.3% of the high-volume centers versus 47.2% of the low-volume centers (*p* = 0.05). The second-generation somatostatin analog (pasiretoid) is chosen in 26% of the high-volume centers and in 14.8% of the low-volume centers (*p* = 0.05). Debulking surgery for an invasive GH-secreting adenoma, in which hormonal remission is not a realistic goal of the surgery and the patient shows no visual deficit, is mostly or always performed in 71.5% of the high-volume and in 51% of the low-volume centers (*p* = 0.01). Some 35.6% of the high-volume centers report the waiting period for surgery as a reason for medical pretreatment in Cushing’s disease. In low-volume centers, this is the case in 15.7% of the centers (*p* = 0.001). In Cushing’s disease, a dynamic MRI is routinely performed (in ≥ 70% of cases) in 55.2% of the high-volume centers and in 40.8% of the low-volume centers (*p* = 0.01). Routine catheterization and petrosal sinus blood sampling are performed in 9.8% of high-volume and 2.9% of the low-volume centers (*p* = 0.05).

### Regions

Surgery is considered a primary treatment modality for prolactinomas in 38.2% of the western centers and in 8.6% of the northern centers (*p* = 0.001). Some 43.2% of the southern centers perform 6–10 GH-secreting adenoma surgeries per year compared to 4.8% in eastern centers (*p* = 0.001). On the other hand, > 10 GH-secreting adenomas are treated in 35.7% of the eastern centers but only 15.5% of the western centers (*p* = 0.001). Inferior petrosal sinus sampling is rarely performed (in ≤ 30% of cases) in 95.1% of the eastern centers and 72.2% of the southern centers (*p* = 0.01).

## Discussion

Surgery for hormone-secreting adenomas is rare in most European neurosurgical centers participating in the survey. Centers performing > 10 cases per hormonal axis were virtually nonexistent. Casanueva et al.^5^ discussed this in their article on the criteria for the definition of a *Pituitary Tumor Center* *of Excellence* (PTCOE). It is not possible to set a limit for each type of hormone-secreting adenoma. Still, the authors support the existence of high-volume centers. They recommend one pituitary center per 2.5–5 million inhabitants. For example, this would support the existence of 16–32 pituitary centers in Germany. Unfortunately, we were not able to cover all the centers performing pituitary surgery. Still, we received completed surveys from 60 centers in Germany. Even more striking data were derived from smaller countries. In all, 13 centers from the Czech Republic participated in the survey and performed pituitary surgery. In all 13 centers from the Czech Republic, participating in the survey, pituitary surgery is performed. The formula of 2.5–5 million Inhabitants per center would call for 2–4 centers in the Czech Republic. Giant and recurrent pituitary adenomas call for the highest experience in pituitary surgery.

Kasper et al. analyzed management of recurrent and residual non-functioning pituitary adenomas in Canada. Endoscopic technique for giant pituitary adenomas resection was more frequently applied by neurosurgeons in practice for less than 10 years. Neurosurgeons who were newer to practice had a greater tendency to advocate for stereotactic radiosurgery or re-resection for residual adenomas^6^.

Zamanipoor Najafabadi et al.^7^ performed a systematic review and meta-analysis on surgery as a viable alternative first-line treatment for patients with prolactinoma. They included 55 articles on medical treatment (*n* = 3564 patients) and 25 on transsphenoidal surgery (*n* = 1836 patients). Long-term disease remission after dopamine agonist withdrawal was 34% and 67% after surgery. Subgroup analysis of microprolactinomas showed 36% disease remission after dopamine agonist withdrawal and 83% after surgery. They concluded that in most prolactinoma patients, disease remission could be achieved through surgery, with low risks of long-term surgical complications. In contrast, disease remission was less often achieved with dopamine agonists. This review may raise questions about whether medical treatment is always the first choice for patients with prolactinoma. Surprisingly, in our survey about 25% of the centers considered surgery as first-line treatment. More often, surgery was seen as a primary treatment modality in western centers than in northern centers.

Timing of pituitary apoplexy surgery in patients on medical therapy for prolactinoma was studied by Rutkowski et al.^8^. In their cohort, 13 patients underwent surgery within 72 h of symptom onset and 19 underwent surgery > 72 h after symptom onset. They concluded that the timing of surgical intervention relative to the onset of symptoms does not significantly affect the resolution of neurological or endocrinological deficits. In addition, early versus delayed resection did not significantly improve visual deficits, total visual loss, resolution of oculomotor palsy, recovery from hypopituitarism, or non-neuroendocrine signs and symptoms such as headache and encephalopathy.

In a study from India, Argawal and Mahapatra^9^ looked at the timing of pituitary apoplexy surgery. The mean delay between apoplexy and neurosurgical consultation was 10 days (range 4–30 days). All patients showing improvement in vision had been operated on within a week of the apoplectic episode. In addition, the authors found that even completely blind eyes might have a remarkable improvement in vision if surgical decompression of the optic apparatus is undertaken early. In our survey, surgery was done within 48 h of onset in 72.1% of centers.

Surprisingly, in 20% of the centers, medical pretreatment was often used in patients undergoing pituitary surgery for acromegaly. If this were the case, then first-generation somatostatin analogs were used most often. This finding is quite surprising given that current guidelines do not recommend routine pharmacological pretreatment for acromegaly^10^. Previously, several studies examined whether presurgical pharmacotherapy could improve endocrinological outcomes. Most of them failed to confirm that pretreatment greatly benefits patients (e.g., a better chance to achieve remission following surgery^11,12^. Others found pharmacotherapy with somatostatin agonists (SSA) beneficial^13^. This could be given by selecting the patients with invasive adenomas or giant adenoma^14,15^. Several studies have shown that treatment with SSA reduces tumor volume^16,17^, suggesting that such an approach might be applied in selected patients. Besides improved endocrine outcome, a positive impact on anesthesia management was considered a possible indication for presurgical pharmacotherapy with SSA. However, this was not confirmed by Losa et al.^12^. Still, presurgical treatment should be considered in selected cases where improvement in some particular complications of acromegaly (e.g., sleep apnea) is the goal. Besides first-generation SSA, another medication is used in the presurgical management of acromegaly. For the other agents (D2 agonist, pasireotide, pegvisomant), reliable data are scarce. However, when the goal is to improve the anesthetic outcome of patients by decreasing GH secretion/action before surgery, these agents can also be used. The impact of using this medication before surgery on long-term endocrine remission is also debatable. When pasireotide was used in presurgical management, no benefit was seen over immediate surgery^18^.

Pretreatment with cortisol lowering medication in patients with Cushing´s is reasonable in severely decompensated individuals in whom improvement of metabolic or cardiac compensation is desired. It has its place when surgery cannot be performed soon and this seems to be a common reason among the centers participating in the survey. Treatment with pasireotide in this setting seems to be a reasonable option because of its dual effect on lowering ACTH and therefore cortisol secretion and its antitumor effect. According to several case reports, it has been successfully used^19^. Treatment with steroid lowering medication (metopirone or ketoconazole) is also widely used in the centers that participated in the survey. It has been confirmed that this management can have a positive impact on surgical outcome with a higher rate of endocrine remission in the group treated with steroid inhibitors^20^.

Serum cortisol monitoring in patients with Cushing´s disease in the days following pituitary surgery to assess surgical outcomes has become a widely accepted practice. It helps predict the endocrinological outcome with a serum cortisol level of < 50 nmol/l as a strong predictor for the remission of the disease with an intermediate range from 50 to 140 nmol/l. However, a decrease in serum cortisol is often delayed. Therefore, repeated measurement on consecutive days with close monitoring of the patient’s status to detect clinical signs or symptoms of cortisol deficiency must be planned. In case cortisol remains high or inappropriately normal (e.g., > 140 nmol/l), early repeated surgery to increase chances of remission of the hypercortisolism should be considered^21–23^. Early reoperation is also less demanding than delayed surgery, which may be more complicated due to fibrosis and adhesions. Reversely, repeated surgery may be associated with a higher incidence of complications, such as permanent or transient diabetes insipidus^21^ and permanent cortisol deficiency^22^. In our survey, half of the centers considered early reoperation if a sufficient cortisol level decline was not observed.

According to Friedman et al.^24^, using a dynamic technique with multiple coronal sequences after intravenous gadolinium injection allows high sensitivity and specificity. A pituitary lesion can be found with this technique in 96% of patients with a biochemical diagnosis of ACTH-dependent Cushing’s disease. In comparison, in suspected Cushing’s disease, a pituitary lesion can be identified with a 50–60% higher diagnostic sensitivity rate than in non-dynamic MRI. Liu et al.^25^ studied a combination of dynamic enhanced MRI and high-dose dexamethasone suppression tests and bilateral inferior petrosal sinus sampling in Cushing’s disease. Some 118 patients with Cushing’s syndrome were included. The positive predictive value of the combined pituitary dynamic MRI and high-dose dexamethasone suppression test was 98.6%, higher than that of catheterization. They concluded that patients with both positive findings in dynamic MRI and high-dose dexamethasone suppression test need no further invasive evaluation to establish a definitive diagnosis of Cushing’s disease. Furthermore, they support the application of bilateral inferior petrosal sinus sampling when negative findings are found in either dynamic MRI or a high-dose dexamethasone suppression test. Surprisingly, only half of the centers in our survey use dynamic MRI routinely in diagnostic algorithms for Cushing’s disease. A dynamic MRI is routinely performed more often in high-volume than in low-volume centers.

De Rotte et al.^26^ demonstrated that more lesions were detected at 7 T than 1.5 T MRI. In five patients, both the 1.5 T and 7.0 TMRI enabled visualization of a lesion on the correct side of the pituitary gland. In three patients, 7.0 T MRI detected a lesion of the pituitary gland, whereas no lesion was visible at 1.5 T MRI. The authors also reported the magnetic susceptibility effect of air in the sphenoid sinus as a potentially disturbing artifact for pituitary gland imaging. Barisano et al.^27^ published a review article on clinical applications for 7 T MRI. They speculated that 7 T MRI might one day become a routine diagnostic technique in patients with MRI negative Cushing’s disease, possibly improving surgical planning and outcomes. However, the current practice in Europe is different, with only two centers (0.8%) in our survey routinely used 7 T diagnostic algorithm for Cushing’s disease.

A consensus statement on Cushing’s diagnosis by Arnaldi et al.^28^ concludes that if the results of clinical, biochemical and radiologic tests are equivocal or discordant, bilateral sampling of the inferior petrosal sinuses must be performed to confirm the presence of a secreting ACTH pituitary adenoma. A narrative review on pituitary magnetic resonance imaging vs. bilateral inferior petrosal sinus sampling was recently published^29^. The authors are convinced that petrosal sinus sampling is an accurate and safe invasive diagnostic method in expert hands and plays an important role within the decisional algorithm for diagnosing and managing Cushing’s syndrome. The same finding was supported by a systematic review and meta-analysis by Wang et al.^30^. They showed that petrosal sinus sampling has a high diagnostic value for detecting source of ACTH over-secretion in patients with ACTH-dependent Cushing’s syndrome. This result contrasts with our findings: catheterization and petrosal sinus blood sampling are rarely or never used in almost 80% of European neurosurgical centers. Routine catheterization and petrosal sinus blood sampling were performed in 9.8% of high-volume centers and in 2.9% of low-volume centers. Petrosal sinus sampling was more frequently performed in southern than in eastern centers.

There is an ongoing debate on the need for routine perioperative corticoid substitution after pituitary adenoma surgery. For instance, Tothi et al.^31^ published a systematic review and meta-analysis on the need for perioperative steroid replacement therapy after pituitary adenoma surgery. They analyzed 18 studies from 11 countries (*n* = 1224 patients) published between 1987 and 2013. The patients with morning serum cortisol levels of < 60 nmol/l at 3 days after operation were considered adrenal insufficiency and > 270 nmol/l as adrenal sufficient. They found that serum cortisol levels had significantly increased in patients after pituitary adenoma surgery. However, there was also significantly increased postoperative adrenal insufficiency and diabetes insipidus in the supplementation group but not in the no supplementation group. Therefore, they concluded that there is no necessity to receive routine cortisol replacement for patients with normal morning serum cortisol levels. Fridman-Bengtsson et al.^32^ evaluated different hydrocortisone treatment strategies in transsphenoidal pituitary surgery. The authors compared three groups: high dose, intermediate dose and low dose hydrocortisone substitution. In the study, all patients received some degree of hydrocortisone substitution. The results indicated that low-dose hydrocortisone therapy favors a better endogenous cortisol production in the early postoperative phase.

De Tommasi et al.^33^ conducted a study on transsphenoidal surgery without steroid replacement in patients with preoperative morning serum cortisol < 250 nmol/l. The results were compared to patients with a preoperative morning serum cortisol > 400 nmol/l and another set of patients with morning serum cortisol < 250 nmol/l who received intraoperative cortisol administration. None of the patients experienced a full syndrome of adrenal insufficiency. One patient with a preoperative morning serum cortisol < 250 mol/l had isolated postoperative fatigue and required cortisol replacement. No patient suffered any life-threatening complications. The authors concluded that pituitary adenoma surgery could be performed safely in patients with preoperative morning serum cortisol < 250 nmol/l in closely monitored settings without intraoperative cortisol administration.

Inder and Hunt published guidelines for perioperative assessment and management of glucocorticoid replacement in pituitary surgery^34^. They recommend that perioperative glucocorticoid substitution should not be considered for patients with intact hypothalamic-pituitary axis function and in whom selective adenomectomy is possible. Early postoperative assessment depends on daily clinical assessment of the patient and morning serum cortisol levels.

Wentworth et al.^35^ performed a prospective evaluation of a protocol for reduced glucocorticoid replacement in transsphenoidal pituitary adenomectomy. A postoperative morning serum cortisol threshold of 250 nmol/l on days 1–3 was used to guide long-term glucocorticoid requirement in the prospective cohort. In two low-risk cases, long-term glucocorticoid replacement was required despite postoperative cortisol > 250 nmol/l. For the remaining 42 low-risk operations, glucocorticoid was not prescribed on hospital discharge based on a morning serum cortisol level of > 250 nmol/l and no clinical evidence of hypocortisolism. Thus, none of these 42 cases required glucocorticoid treatment for hypocorticolism following surgery.

All the studies mentioned above may call for selective hydrocortisone substitution in patients with no preoperative hypopituitarism and selective adenoma surgery. Still, in almost 60% of the centers participating in our survey, a routine dosage of hydrocortisone substitution was administered.

### Study limitations and strengths

The main limitations of our study are related to study design. Every survey suffers from sampling bias due to nonresponse, i.e. nonresponse impacts the representativeness of the survey results, causing estimation bias. In addition, the quality of responses relies on the integrity of the respondents. We also failed to obtain a high response rate from some European countries (see results). Most results came from Germany, Italy, UK, Czech Republic and Spain. We achieved a large sample of data with a very high response rate and 100% completion rate. We want to emphasize that every respondent in our survey represents one neurosurgical center. So far, this study is the largest survey on pituitary adenoma surgery in Europe.

## Conclusions

Our survey shows that in most centers, few hormone-secreting adenomas are treated per year. In about 25% of the centers, prolactinoma surgery may be regarded as first-line treatment; in about 20% of the centers, medical treatment is the first-line treatment for GH-secreting adenomas. Pretreatment for Cushing’s disease is routinely used in 21% of centers. Surgery for GH-secreting adenomas was performed in 60% of the centers if hormonal remission was not realistic due to invasiveness of the adenoma, and the patient had no visual loss. Dynamic MRI was routinely used for Cushing’s disease in about 50% of the centers. In contrast, petrosal sinus blood sampling and 7 T MRI were seldom used. Routine hydrocortisone substitution was applied in 60% of the centers.

## Ethics approval

Approved by Ethical Committee of Central Military Hospital, Prague, Czech Republic.

## Consent to participate

Informed consent was obtained from the participants.

## Consent for publication

All the members of study team consent with publication.

## Supplementary Information

Supplementary Information.

## Data Availability

All raw data are available upon request.
